# The Development of Foodborne Pathogen Detection and Biosensor Design for Surface Plasmon Resonance Technology

**DOI:** 10.3390/bios15120774

**Published:** 2025-11-25

**Authors:** Ye Hu, Jun Yang, Jian Chen, Xiaojie Sun, Wenyan Hu, Xinmei Liu

**Affiliations:** 1 Key Laboratory of Detection and Traceability Technology of Foodborne Pathogenic Microorganisms, State Administration for Market Regulation, Nanjing 211198, China; 2 Nanjing Institute for Food and Drug Control, Nanjing 211198, China; 3 Key Laboratory of Detection and Traceability Technology of Foodborne Pathogenic Bacteria for Jiangsu Province Market Regulation, Nanjing 211198, China; 4 School of Chemistry and Chemical Engineering, Hunan University of Science and Technology, Xiangtan 411201, China

**Keywords:** foodborne pathogens detection, biosensor design, surface plasmon resonance

## Abstract

The rapid detection of pathogenic bacteria is important for the global public health field. Existing detection technologies are generally limited by log efficiency, high costs, and susceptibility to contamination. Advanced detection methods, such as surface plasmon resonance (SPR), have been proposed to break these limitations, with the advantages of fast detection speed and high sensitivity. SPR operates on the principle of attenuated total internal reflection at a metal–dielectric interface. This technique exploits the resonant absorption of incident photons by surface plasmons, facilitating the detection of tiny variations in the local refractive index by tracking the resonance condition change. This review provides a comprehensive overview of the development in the detection of foodborne pathogens using SPR technology, including a detailed discussion of the working principles of SPR, related instrumentation, and various detection methods. Specifically, this review focuses on a discussion of different SPR detection methods in terms of assembly processes, detection specificity, sensitivity, and detection limits in SPR biosensors, aiming to provide the readers with a fundamental knowledge of SPR.

## 1. Introduction

With the rapid development of the economy and society, food safety issues have become increasingly prominent, particularly the contamination of foodborne pathogens, which threaten public health [1,2]. These pathogenic microorganisms are easily transmitted through food, leading to various diseases. Symptoms in mild cases include headache, fever, and diarrhea, while severe cases can lead to cancer, organ damage, or even death. In addition, the toxic metabolites (mycotoxins) produced by fungi can also threat food safety [3]. More than 400 mycotoxins have been identified, such as aflatoxin B1 (AFB1), ochratoxin A (OTA), and patulin (PAT), which possess a high toxicity and wide contamination range, posing severe threats to human safety [4]. Therefore, the efficient detection and control of mycotoxin contamination plays a crucial role in food safety. The misuse of antibiotics has led to a rapid increase in antibiotic-resistant strains, further complicating disease treatment [5]. In addition, against the backdrop of the worsening natural environment, the frequent occurrence of emerging diseases and zoonotic infections has highlighted the increasing importance of the rapid detection of pathogenic microorganisms. Specifically, hundreds of millions of people worldwide are affected by foodborne diseases each year [6], which clearly highlights the severe results caused by foodborne pathogens and also indicates the importance of pathogen detection.

*Escherichia coli* O157:H7, *Salmonella* spp, and *Listeriamonocytogene* are regarded as the three most significant foodborne pathogens due to their severe health effects and substantial social impact [7,8]. *Escherichia coli* O157:H7 first gained widespread attention in the United States due to a large-scale outbreak of hemorrhagic colitis [9]. Even at a low dose, this bacterium can cause hemorrhagic diarrhea and severe enteritis, resulting in significant medical and socio-economic burdens. *Salmonella* can also lead to foodborne outbreaks. In 2012, 25 states in the United States experienced successive *Salmonella* outbreaks [10]. The frequent occurrence of *Salmonella* infections severely threatens food safety and public health systems. *Listeriamonocytogenes* is a typical zoonotic pathogen, with mild infections presenting as gastroenteritis, while severe cases can lead to conditions such as sepsis, meningitis, and monocytosis [11]. Although its incidence is lower compared to other pathogens, its fatality rate is as high as 30–40%, far exceeding that of other common foodborne pathogens. Therefore, strengthening research on the rapid and efficient analytical detection of these pathogens is of significant importance for ensuring food safety and public health.

However, existing traditional detection methods have many shortcomings in practical applications, necessitating technological innovations to meet the high standards required for food safety testing [12]. The traditional plate culturing method is considered the conventional “gold standard” [13], but this method typically has low efficiency due to its long processing time and high technical requirements for media preparation and colony counting. Polymerase chain reaction (PCR) is based on the principle of nucleic acid base complementary pairing [14]. It involves designing specific primers and extracting, amplifying, and ultimately detecting target genomic DNA using fluorescence quantification or gel electrophoresis. PCR can identify specific pathogens with high sensitivity. However, PCR testing is costly, relies on expensive instruments and equipment, involves complex operations, and is not suitable for rapid on-site detection. Therefore, developing efficient, rapid, sensitive, and easy-to-operate detection technologies has become a research hotspot. This review systematically compares the principles and characteristics of existing technologies for detecting foodborne pathogens and provides an in-depth analysis of their respective advantages and limitations (Table 1). In contrast, SPR technology can detect foodborne pathogens in a label-free and real-time way. Due to its operational simplicity, high detection sensitivity, and strong real-time monitoring capability, SPR technology has shown significant advantages in fields such as protein interaction analysis, DNA molecular hybridization, ligand–receptor interactions, and small molecule drug design [15]. Currently, various commercial SPR instruments are available worldwide, such as those produced by Biacore AB (Uppsala, Sweden), Windsor Scientific (Slough, UK), and Texas Instruments (Dallas, TX, USA), which have strongly advanced the development of detection and diagnosis [16]. In the context of new public health challenges, the development of SPR will continue to benefit public health to a widespread extent.

This review aims to provide a systematic overview of recent advances in SPR technology for detecting foodborne pathogens. We begin with the technical fundamentals of SPR, followed by a classification of the primary detection methodologies (direct vs. indirect). Subsequently, we delve into the key technological advances driving performance improvements, with a particular focus on material engineering, surface functionalization strategies, and advanced sensor designs. Finally, we discuss the current advantages and limitations of SPR in this field and offer perspectives on future developments.

## 2. The Technical Fundamentals of SPR Technology

**Figure 1:**
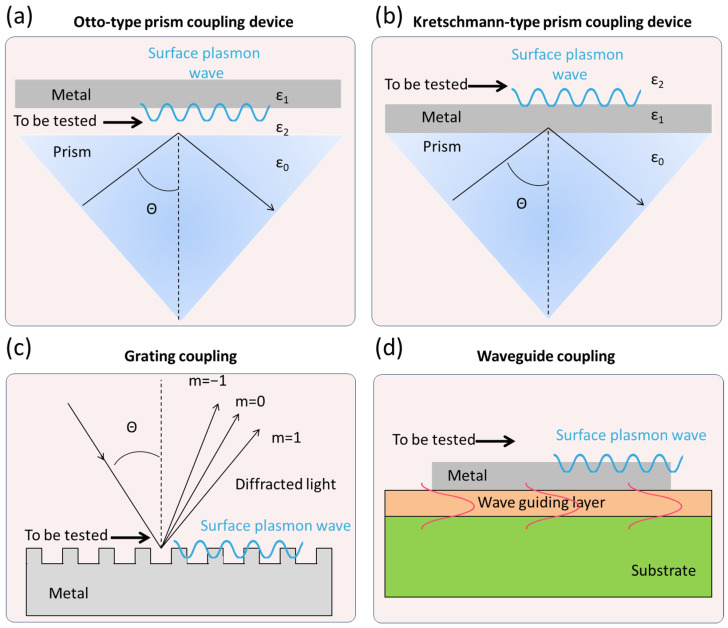
The commonly used coupling devices of SPR. The black lines represent incident light and reflected light, respectively; the blue lines represent surface plasmon wave; the orange waves represent guided mode.

The key to SPR technology lies in the SPR optical wave coupling mechanism. Specific coupling devices are required to achieve the coupling of optical waves with surface plasmons and generate the SPR signal. The most commonly used coupling devices can be classified into prism coupling, grating coupling, and waveguide coupling (Figure 1). In the Otto-type prism coupling device, a gap is maintained between the bottom surface of the prism (ε_0_) and the metal film (ε_1_), where the substance to be tested (ε_2_) flows through [24]. When the incidence angle is over the critical angle, total internal reflection occurs at the ε_0_/ε_2_ interface, which interacts with the resulting evanescent wave to excite SPR. In the Kretschmann-type prism coupling device, the bottom of the prism is attached to a metal film that is tens of nanometers thick while the substance to be tested is placed on the other side of the metal film. The evanescent wave passes through the metal film and interacts with the ε_1_/ε_2_ interface to excite SPR [25]. The SPR signal strength depends critically on the metal film’s thickness: a thick film leads to undue decay of the evanescent wave before it reaches the ε_1_/ε_2_ interface, whereas a thin film results in a weakened resonance effect. The Kretschmann-type device is widely used in modern SPR instruments due to its simple structure and ease of operation. Grating coupling occurs when the incident light strikes a periodically modulated metal grating, causing diffraction of the reflected light, with different diffraction angles corresponding to different diffraction orders [26]. The SPR effect is generated if the wave vector of a particular diffracted light matches that of the surface plasmon wave. Grating coupling requires a highly periodic structure in the grating but offers greater flexibility. Waveguide coupling involves the propagation of optical waves through a waveguide layer with micrometer-scale width [24]. When the light passes through the area covered by a metal film, it generates an evanescent wave. The evanescent wave can penetrate the metal film to induce the SPR effect at its surface with the surrounding dielectric. This coupling method is suitable for portable devices.

**Figure 2:**
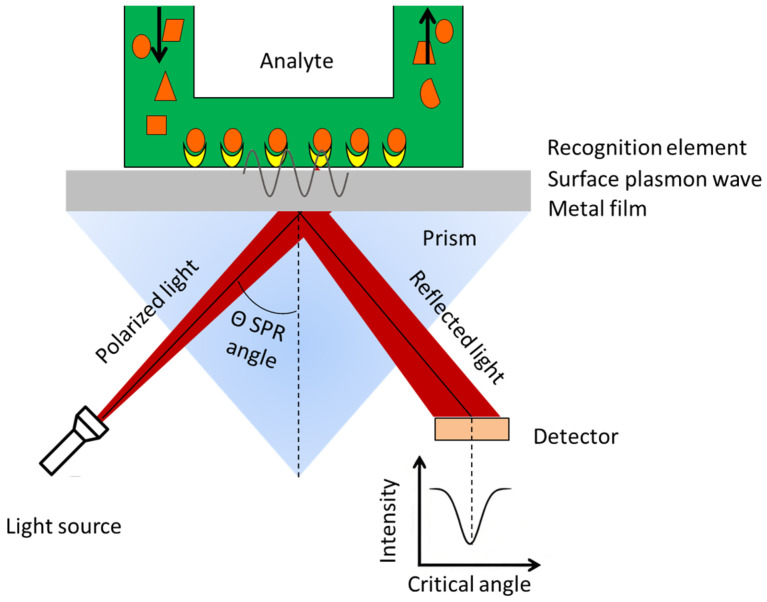
The schematic diagram of SPR. Arrows indicate the flow direction of analyte. Orange shapes indicate the analyte and the yellow shapes represent the biorecognition element.

The research on SPR technology began in the early 20th century, when Wood and others first discovered the SPR phenomenon while studying the anomalous diffraction phenomenon of diffraction gratings [27]. In 1909, Sommerfeld and others, based on Maxwell’s electromagnetic theory, introduced the concept of the complex dielectric constant, providing a theoretical explanation for the electromagnetic waves confined to the vicinity of a surface [28]. Later, Ritchie observed experimentally that high-energy electrons passing through a metal film experience energy loss at the plasma frequency, further deepening the understanding of SPR [29]. In 1960, Stern and Farrell pointed out that the presence of a metal–dielectric interface is a necessary condition for SPR to occur, because the transverse magnetic (TM) wave needs to propagate along the metal–dielectric interface, with their amplitude exponentially decaying as the distance from the interface increases [30]. This plasmonic oscillation phenomenon is confined to the surface or interface region. Subsequently, scholars such as Otto and Kretschmann further studied the mechanism of optical excitation of SPR, laying the experimental foundation for SPR technology [30]. During the detection process, the substances on the surface of the metal sensor chip can react with the target substance to alter the refractive index at that location, thereby causing a shift in the resonance wavelength or resonance angle and ultimately leading to a change in the SPR signal intensity (Figure 2). Qualitative and quantitative detection of the target substance can be measured by monitoring the changes in the SPR signal.

## 3. Classification of SPR Methods for Detecting Foodborne Pathogenic Bacteria

Building upon the theoretical discussion in previous sections, SPR-based detection of pathogenic bacteria can be broadly categorized into two main approaches: the direct method and the indirect method (also known as the subtractive inhibition method). Each strategy leverages the principles of SPR in distinct ways to achieve sensitive and specific pathogen identification, with the choice of method often depending on the target analyte, sample matrix, and desired detection limits [31].

### 3.1. Direct Detection

**Figure 3:**
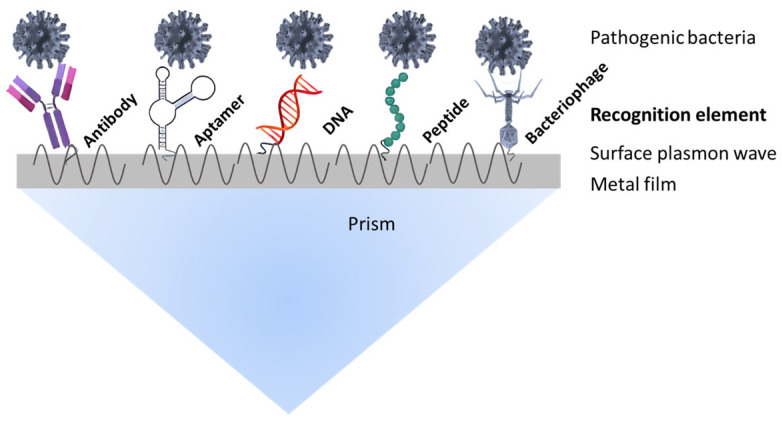
SPR technology based on the binding of specific ligands on the chip surface for pathogenic bacteria detection.

In the direct detection method, SPR is employed to monitor the real-time binding interactions between pathogenic bacteria and specific recognition elements immobilized on the sensor surface. This method relies on the specific affinity of ligands such as antibodies, nucleic acid probes, aptamers, or peptides for their bacterial targets (Figure 3). The SPR response signal variation directly correlates with pathogenic bacterial mass, enabling quantitative analysis without the need for secondary labeling or amplification steps [26,32]. The amplitude of the SPR signal thus reflects the bacterial concentration, providing a straightforward and rapid detection platform [33,34].

#### 3.1.1. Detection Using Antibodies

Antibody detection is based on immunological interactions, specifically the binding between antigens and antibodies, which forms a stable complex due to the highly complementary nature of the epitopes of antigens and the hypervariable regions of antibodies. These interactions have two main characteristics: Specificity: The spatial complementarity between the antigen epitopes and the hypervariable regions of the antibody molecules ensures the high selectivity of the binding, allowing the antibody to specifically recognize the target pathogenic bacteria. Reversibility: following complex formation, the binding can dissociate under specific conditions, restoring the antigen and antibody to their free states. This reversibility provides antibody detection with the ability to regenerate the sensor. Capitalizing on their outstanding specificity and reversible binding characteristics, antibodies constitute one of the most essential biosensing components in SPR systems, making immunoassays a dependable strategy for foodborne pathogen detection. In recent years, many researchers have also actively explored approaches to enhance the performance of antibody detection.

Detection strategies based on nanomaterial enhancement

**Figure 4:**
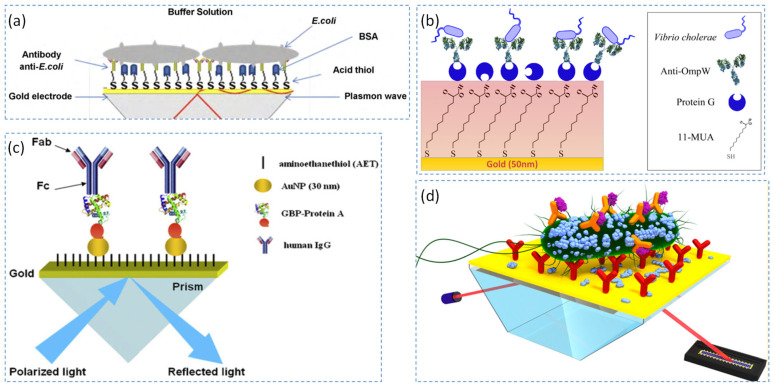
The representative antibody detection strategies based on SPR technology, such as an SPR biosensor using anti-*E. coli* for the detection of *E. coli* bacteria (**a**) [36], an SPR biosensor using anti-OmpW for the detection of *Vibrio cholerae* (**b**) [44], an SPR biosensor using anti-*Salmonella* antibodies for the detection of *Salmonella typhimurium* (**c**) [47], and an SPR biosensor using mouse monoclonal anti-*Salmonella* antibody ab8274 for the detection of *Salmonella* in powdered milk (**d**) [55].

Nanomaterials have been widely incorporated into SPR biosensors to enhance their detection performance owing to their unique physicochemical properties, which significantly improve sensitivity and specificity [35]. Baccar et al. developed two distinct bacterial biosensors [36]. One sensor is a gold substrate functionalized with acid–thiol, and another sensor is created through the immobilization of gold nanoparticles onto a modified gold surface. Gold nanoparticles were employed to enhance the biosensor’s sensitivity by increasing the surface area-to-volume ratio, thereby improving sensitivity and lowering the detection limit compared to bulk gold surfaces (Figure 4a). Wang et al. also developed a gold nanoparticle/Au film-enhanced optical fiber SPR biosensor, which exhibited a linear detection range of 10^2^–10^8^ CFU/mL and a detection limit of ~50 CFU/mL [37]. Likewise, by using ultra-low fouling and functionalizable poly, Vaisocherová-Lísalová et al. developed an SPR biosensor with a detection limit of 17 CFU/mL for *E. coli* in cucumber and 7.4 × 10^3^ CFU/mL for *Salmonella* spp. in hamburger [38].

Beyond noble metal nanostructures, Kaushik et al. developed a fiber optic SPR immunosensor enhanced with MoS_2_ nanosheets for detecting *Escherichia coli* (*E. coli*). The MoS_2_ nanosheets were first integrated onto a gold surface, creating a functionalized platform for the sensor. Monoclonal antibodies targeting *E. coli* were subsequently immobilized on the MoS_2_-coated surface via hydrophobic interactions. The presence of MoS_2_ nanostructures significantly improved the sensor’s sensitivity, enabling the detection of *E. coli* at a low limit of 94 CFU/mL [39].

Furthermore, magnetic nanomaterials offer unique advantages for target separation. Liu et al. developed an SPR biosensor using antibody-functionalized Fe_3_O_4_ immunoMNPs as probes to selectively capture *Salmonella* enteritidis from complex sample matrices under an external magnetic field. The immunoMNPs not only acted as transporters to rapidly deliver the analyte to the sensor surface, but also functioned as labels of the refractive index change enhancers detected by SPR. Therefore, this biosensor demonstrated a remarkable sensitivity, detecting S. enteritidis at concentrations as low as 14 CFU/mL, with a linear detection range from 1.4 × 10^1^ to 1.4 × 10^9^ CFU/mL [40].

Protein-based oriented immobilization and antibody conjugation strategies

Recently, numerous SPR technologies have focused on protein-based oriented immobilization and antibody conjugation strategies to enhance detection performance. Morlay developed an immuno-chip SPR imaging technique, producing specific antibodies against *Listeria* that were chemically grafted onto a biochip. These antibodies were then tested for their ability to distinguish between different *Listeria* strains. The resulting antibodies exhibited strong performance, particularly, the Ab6 and Ab4 immunoglobulins, which effectively recognized *Listeriamonocytogenes* in both pure cultures and food samples. Additionally, a polyclonal antibody, Ab2, demonstrated specificity for other *Listeria* species, offering a versatile tool for pathogen detection [41].

Meanwhile, multiple groups have utilized protein G as a universal scaffold for antibody orientation. For instance, Bae et al. constructed an imaging ellipsometry-based immunosensor using a layered architecture of 11-mercaptoundecanoic acid (11-MUA), protein G, and a monoclonal antibody (Mab) specific to *L. pneumophila*, achieving detection down to 10^3^ CFU/mL [42]. Oh et al. adopted a similar strategy, covalently linking protein G to an 11-mercaptoundecanoic-modified gold surface using EDAC, followed by antibody attachment, which allowed the detection of *L. pneumophila* at 10^2^ CFU/mL [43]. The same protein G-based immobilization approach was also applied to cholera detection. Taheri et al. designed an SPR-based immunosensor for the precise detection of *Vibrio cholerae* by utilizing anti-OmpW antibodies. To facilitate antibody immobilization, protein G was covalently attached to an 11-mercaptoundecanoic acid (11-MUA) surface using amine coupling. The anti-OmpW antibody was then oriented and immobilized onto the protein G layer through bioaffinity interactions. Thanks to the strong binding affinity between OmpW and its specific antibody, the immunosensor achieved a remarkable detection limit of 43 cells/mL, demonstrating its high sensitivity for detecting *Vibrio cholerae* [44] (Figure 4b). Likewise, Jyoung et al. developed a SPR immunosensor to detect *Vibrio cholerae O1* by functionalizing the sensor surface with a protein G layer, and extended the detection range from 10^5^ to 10^9^ cells/mL [45].

Similarly, in research on *Salmonella*, Hyeon et al. cloned and purified the complete tail protein of Det7 and assessed its selective binding properties to *Salmonella typhimurium* and *Escherichia coli* K-12 using microagglutination assays and transmission electron microscopy (TEM). The protein showed strong affinity for its natural host, *S. Typhimurium*, while exhibiting no binding to its non-host, *E. coli* K-12, highlighting its selectivity. Following this, they immobilized the Det7T protein on gold substrates to develop an innovative SPR biosensor aimed at detecting *Salmonella enterica serovar Typhimurium* [46]. Complementing this effort, Ko et al. designed a fusion protein combining gold-binding polypeptides (GBP) and protein A (ProA) to improve antibody immobilization efficiency. The GBP-ProA was self-assembled on gold surfaces, enabling oriented binding of antibodies via their Fc regions, which streamlined the construction of the immunosensor for detecting *Salmonella typhimurium* without complex surface chemical modifications [47] (Figure 4c).

For the detection of *Mycobacterium tuberculosis*, Trzaskowski et al. employed antibodies against the Ag85 protein—a major secretory antigen of the bacterium. Immobilized on sensor chips via carboxyl group and amine coupling techniques, these antibodies enabled detection of Ag85 at concentrations as low as 10 ng/mL, highlighting the potential of protein-specific capture in clinical samples [48].

Surface modification strategies employing self-assembled monolayers (SAMs)

Surface modification strategies employing self-assembled monolayers (SAMs) have been widely adopted to improve SPR-based biosensors’ performance. Lin et al. developed an immunosensor that integrated side-polished optical fiber with SPR for detecting *Legionella pneumophila*. The optical fiber was side-polished to expose half of its core. The exposed surface was then covered by a thin 37 nm gold film, which was covered by a SAM of 11-mercaptoundecanoic acid (MUA) to facilitate the immobilization of antibodies. The antibodies specific to *L. pneumophila* were then attached to the MUA-modified surface, enabling the detection of antigen binding. The SPR response from this antigen–antibody interaction allowed the sensor to achieve a detection limit of 10 CFU/mL [49].

Similarly, Puttharugsa et al. explored a mixed strategy, using self-assembled monolayers in conjunction with SPR imaging for the detection of *Acidovorax avenae* subsp. *Citrulli* (*Aac*), a seed-borne bacterium. The monoclonal antibody 11E5 was immobilized on the mixed SAM surface, achieving a detection limit of approximately 10^6^ CFU/mL for *Aac* [50]. Yodmongkol et al. introduced a novel SPR imaging approach for detecting *Candida albicans*, employing both direct and sandwich antibody-based detection methods. The efficacies of both detection strategies were evaluated in a mixed microbial suspension, revealing a detection limit of 10^7^ cells/mL for the direct method and 10^6^ cells/mL for the sandwich assay. This comparative analysis demonstrated the enhanced sensitivity of the sandwich approach [51]. Furthermore, Zhang et al. developed a multichannel SPR biosensor that simultaneously detected *E. coli* O157:H7, *Salmonella enteritidis*, and *Listeria monocytogenes* in food samples with a low detection limit of 14 CFU/mL [52].

Innovative SPR Sensing Strategies and Surface Functionalization Methods

Meneghello et al. developed an SPR-based device for detecting *Legionella pneumophila*, utilizing highly sensitive azimuthally controlled grating coupling. This configuration enabled the detection of resonance wavelength shifts spanning from 46.3 nm at 10^6^ CFU to 2.46 nm at 10 CFU. The device demonstrated a sensitivity up to 1000 times higher than conventional fluorescence assays, making it an exceptionally effective tool for *L. pneumophila* detection [53].

In surface functionalization research, Makhneva et al. developed a nitrogen-functionalized surface via plasma polymerization for SPR immunosensing. They deposited plasma polymers (PPs) onto gold sensor surfaces using cyclopropylamine (CPA) vapors. The resulting surface was then activated with glutaraldehyde (GA) to provide a stable platform for antibody immobilization. This method produced immunosensors with a detection limit of 10^5^ CFU/mL, demonstrating its potential for sensitive bacterial detection. [54]. To further enhance detection sensitivity through signal amplification, Farka et al. developed an enzymatic precipitation-enhanced SPR immunosensor for *Salmonella Typhimurium* and achieved a detection limit of 100 CFU/mL and a linear range of up to 10^6^ CFU/mL, markedly improving sensor performance [55] (Figure 4d).

Comparative Evaluation of SPR Sensing Methodologies

Several research groups have conducted systematic comparisons of different SPR-based sensing strategies to identify optimal approaches for bacterial detection, evaluating their performance in terms of sensitivity, efficiency, and practical applicability. Bhandari et al. evaluated three SPR detection strategies for lettuce samples contaminated with Salmonella typhi at varying concentrations: (1) a direct detection method, where monoclonal antibodies were immobilized on sensor surfaces to capture the bacterial flagellin; (2) a two-step sandwich method, involving initial direct detection followed by antibody-mediated sandwich formation on captured flagellin; and (3) a pre-incubation one-step sandwich method, where monoclonal antibodies were pre-incubated with the sample prior to detection [56]. In a similar comparative approach, Torun et al. compared four different SPR-based sensing techniques for *Escherichia coli* detection by evaluating their linear ranges, detection limits, and response times [57]. Therefore, the use of antibodies as recognition elements can effectively enhance the detection performance of SPR sensors for foodborne pathogenic bacteria. Furthermore, by integrating nanomaterials with advanced surface modification techniques, the interfacial properties of the sensor can be precisely controlled, thereby significantly improving detection specificity, lowering the detection limit, and expanding the range and variety of pathogenic bacteria that can be accurately detected.

#### 3.1.2. Detection Using Aptamers

**Figure 5:**
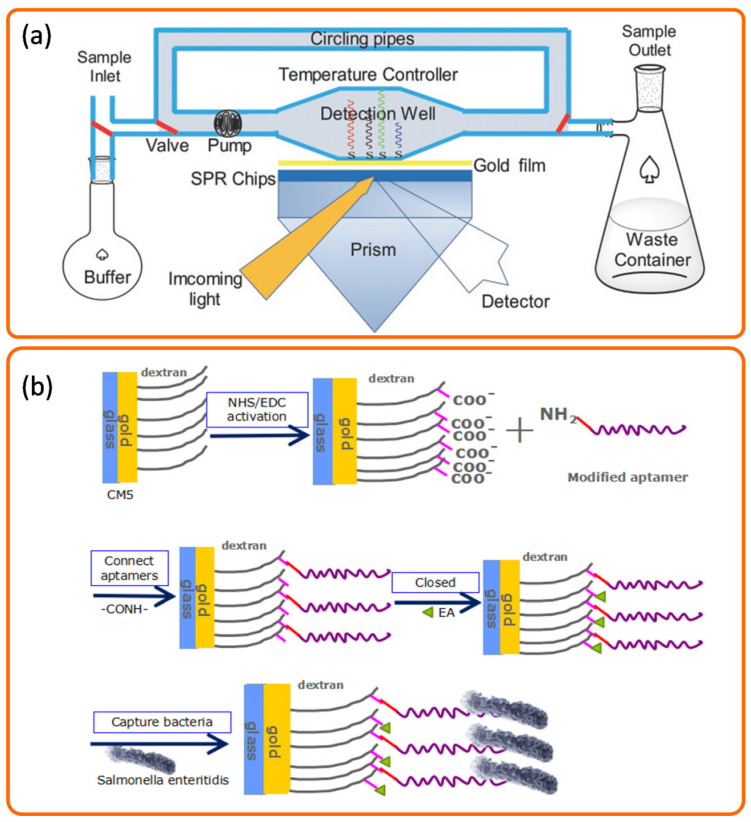
Representative aptamer detection strategies based on SPR technology, such as an SPR biosensor using specific nucleic acid probes for the detection of mixed infections of four pathogenic microorganisms (**a**) [58] and anSPR biosensor using a nucleic acid aptamer as an immune recognition element for the detection of *Salmonella enteritidis* in food (**b**) [61], grey lines represent dextran and purple lines represent aptamer.

As biosensing elements, aptamers exhibit high-affinity binding to specific targets, with recognition capabilities comparable to antibodies; hence, they are often referred to as “chemical antibodies”. Compared to antibodies, nucleic acid aptamers reveal high stability, sensitivity, purity, and regeneration capability, making them suitable for the detection of foodborne pathogenic bacteria. Wang et al. introduced a rapid, label-free approach for detecting mixed bacterial infections using SPR. In their method, aptamers from four pathogenic microorganisms (*Pseudomonas aeruginosa*, *Staphylococcus aureus*, *Clostridium tetani*, and *Clostridium perfringens*) were simultaneously extracted and amplified with universal primers. The resulting single-stranded amplicons were hybridized with a thiol-functionalized probe, which was immobilized on a multi-channel gold (Au) chip through an Au-S bond [58] (Figure 5a). Wang et al. developed an SPR-based aptasensor for specific detection of *Escherichia coli* and *Staphylococcus aureus* using aptamers (37–88 nucleotides) as recognition elements. These aptamers were immobilized on a gold substrate via a polyadenine-mediated approach, enabling precise control over aptamer density through adjustment of salt type and concentration (e.g., NaCl or NaBr). The sensitivity was further enhanced by kinetic SPR measurements to monitor bacterial binding events, resulting in an efficient platform for bacterial detection [59].

To detect the cereal pathogen *Fusarium culmorum*, Zezza et al. developed an SPR sensor based on DNA hybridization. Their method began by amplifying a specific DNA fragment via PCR, from which a 25-mer oligonucleotide probe was designed and immobilized on the sensor. The study further investigated how denaturing agents and ionic strength affected the hybridization efficiency of double-stranded PCR products with this probe, and also evaluated the probe’s specificity [60]. While antibodies are highly specific, they suffer from drawbacks such as high costs and long preparation times. To overcome these limitations, Di et al. developed a label-free and non-destructive detection method based on surface plasmon resonance (SPR) technology. The research team employed aptamers—single-stranded DNA or RNA molecules—which are favored for their high specificity, targeting versatility, ease of synthesis, good stability, and convenient storage. The aptamers were utilized as the recognition element in the SPR biosensor. Carboxymethylated dextran CM5 chips were selected as the sensing substrate, and the aptamers were immobilized on the chip surface via an amide bond, ultimately achieving effective detection of *Salmonella enteritidis* [61] (Figure 5b).

Moreover, Daems et al. introduced a novel fiber optic surface plasmon resonance (FO-SPR) technique, which offered the capability to simultaneously quantify and identify multiple DNA targets on a single platform. This method leveraged the fact that hybridization probes induced complementary complex accumulation on the sensor surface while DNA targets duplicated during the PCR amplification process. In each subsequent thermal cycle, the DNA target strands dissociated from these probes, leading to the release of gold nanoparticles (AuNPs) from the FO-SPR surface. This release resulted in a change in the mass on the sensor, which in turn altered the refractive index. These shifts in the refractive index provided valuable data for determining the characteristic melting temperature (Tm) of the target DNA sequence, thus enabling its precise identification. The experimental results demonstrated that the developed FO-PCR-M system achieved the same level of resolution as conventional singleplex assays, confirming its capability to distinguish between two bacterial targets in a single sample [62]. Therefore, in addition to antibodies, aptamers—represented by DNA or RNA molecules—constitute another important and emerging class of recognition elements. When combined with SPR technology, aptamers can effectively overcome the inherent limitations of traditional antibodies, such as susceptibility to denaturation, poor stability, stringent storage requirements, high production costs, and limited modification flexibility. Consequently, aptamer-based SPR sensing technology is regarded as a promising and significant research direction for future detection of foodborne pathogenic bacteria.

#### 3.1.3. Detection Using Bacteriophage

Beyond nucleic acid aptamers, bacteriophages—viruses that specifically infect bacteria and other microorganisms—have also emerged as promising recognition elements for SPR biosensing. These non-cellular entities typically contain only one type of nucleic acid and exhibit high specificity in bacterial capture. Due to their selective binding ability, bacteriophages can be employed in SPR biosensors for efficient and accurate detection of foodborne pathogens, offering a novel technological approach to food safety monitoring. For instance, Karoonuthaisiri et al. explored the application of filamentous M13 bacteriophages expressing short peptides as alternative binding agents in an SPR-based sensor for detecting *Salmonella*. The performance of this bacteriophage-based SPR sensor was highly dependent on assay conditions, including immobilization, interaction, and regeneration buffers. With optimized conditions, the bacteriophage-based SPR assay achieved a detection limit of 8.0 × 10^7^ CFU/mL for *Salmonella*, providing a novel approach for bacterial detection [63]. Therefore, compared with traditional recognition elements, bacteriophages—an emerging class of biological recognition agents—offer a new avenue for constructing highly specific SPR sensors for foodborne pathogenic bacteria. Owing to their strict host specificity, bacteriophages enable precise bacterial identification and typing, thereby providing a powerful strategy for developing next-generation, high-specificity SPR detection platforms.

### 3.2. Indirect Detection

#### Subtractive Inhibition Detection

The fundamental principle of the subtractive inhibition method entails combining antibodies of known concentrations with the target bacteria and separating the unbonded ones. Subsequently, an antigen specific to the target bacteria is coupled to the chip. The binding between the free antibodies and the antigens on the chip induces an SPR response signal. By detecting the concentration of the free antibodies, the concentration of the target bacteria can be inversely calculated, thus achieving the indirect detection of the target bacteria. A key advantage of this method lies in its significant signal amplification, which improves detection sensitivity and lowers the detection limit. Subtractive inhibition-based SPR biosensors effectively overcome the sensitivity constraints of direct SPR detection and avoid the complexities associated with monoclonal antibody production. Furthermore, this strategy outperforms conventional antibody-based methods in distinguishing between live and dead bacteria, offering a promising solution for accurate detection of foodborne pathogens.

To overcome the limited penetration depth of the evanescent wave—a major challenge in the SPR-based detection of large pathogens—Masdor et al. developed a subtractive inhibition assay (SIA) for the rapid detection of *Campylobacter jejuni*. In this method, a sample containing *C. jejuni* was first incubated with specific rabbit polyclonal antibodies. After removing unbound antibodies via centrifugation, the mixture was injected over an SPR sensor chip immobilized with secondary goat anti-rabbit IgG antibodies. This approach effectively captures the antibody-bound bacterial cells, circumventing the sensitivity loss typically caused by the large size of pathogen–antibody complexes in conventional sandwich assays. The SIA format mitigates this issue by detecting only the free antibodies, leading to improved sensitivity. The SIA-SPR system developed by Masdor et al. achieved a notably low detection limit of 131 ± 4 CFU/mL for C. *jejuni*, demonstrating the utility of this approach for highly sensitive pathogen monitoring [64]. At the same time, we selected several representative SPR sensors that have been applied to the detection of foodborne pathogenic bacteria and conducted a comparative analysis of their detection limits, linear ranges, and the types of pathogens detected (as shown in Table 2). The results indicate that some sensors can achieve detection limits as low as 1 CFU/mL, while others exhibit a relatively wide detection range (for example, 1.4 × 10^1^–1.4 × 10^9^ CFU/mL). These findings demonstrate that by integrating recognition elements such as antibodies, aptamers, and bacteriophages with nanotechnology, SPR sensors can effectively detect foodborne pathogenic bacteria with both low detection limits and broad linear ranges.

## 4. Research Progress on SPR Technology in the Detection of Foodborne Pathogens

The classification of SPR detection methods into direct and indirect approaches provides a framework for understanding the assay design. However, the performance of any method—whether direct or indirect—is ultimately determined by the sophistication of its underlying technology. Recent progress, particularly in nanomaterial engineering, surface chemistry, and sensor design, has been instrumental in pushing the boundaries of sensitivity, specificity, and practicality.

### 4.1. Nanomaterial Integration and Heterostructure Design for Enhanced SPR Performance

**Figure 6:**
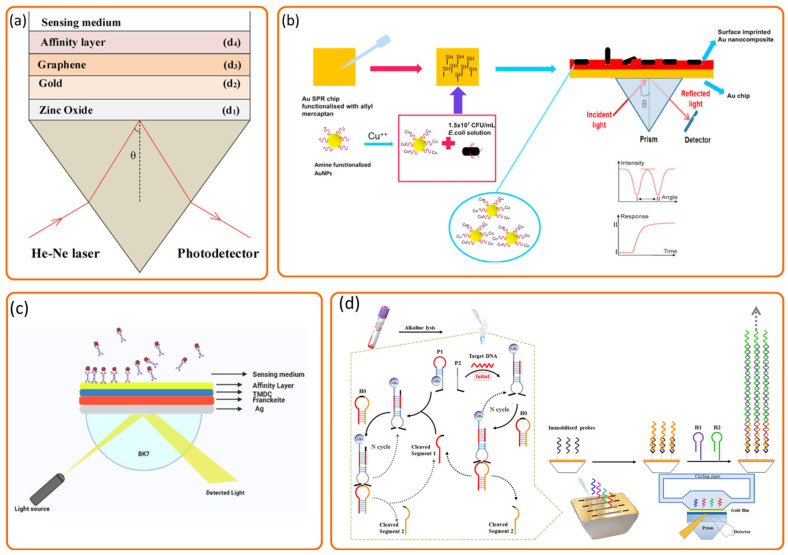
Representative direct detection strategies based on SPR technology, such as an SPR biosensor using zinc oxide, gold, and graphene for the detection of *pseudomonas*-like bacteria (**a**) [69], an SPR biosensor using surface-imprinted plasmonic nanoscale for the selective detection of *Escherichia coli* (**b**) [65], an SPR biosensor using a heterostructure of franckeite and TMDCs for *Pseudomonas* bacteria detection (**c**) [70] and an SPR biosensor using autocatalytic MNAzyme for the simultaneous detection of bacteria from nosocomial bloodstream infection specimens (**d**) [67].

Functional optimization of sensors using various nanomaterials is an effective method for enhancing detection capabilities, enabling efficient bacterial capture and signal transduction. Gasparyan et al. demonstrated the successful application of anisotropic silver nanoparticles for the quantitative detection of bacterial species using two distinct SPR bands, and the achieved detection limit was 1.5 × 10^4^ CFU/mL for *Staphylococcus aureus* [68]. In a theoretical study, Kushwaha designed a ZnO/Au/graphene-based SPR biosensor for *Pseudomonas* detection that achieved a sensitivity of 187.43 °/RIU and a detection accuracy of 2.05 deg^−1^ [69] (Figure 6a).

Beyond the application of individual nanomaterials, the rational design of specialized structures has emerged as a powerful strategy to further augment sensor performance. Janze et al. developed an SPR biosensor for detecting Pseudomonas bacteria using a structure incorporating franckeite, transition metal dichalcogenides (TMDCs; MoS_2_, MoSe_2_, WS_2_, WSe_2_), and a silver layer. The TMDCs were integrated to boost light absorption and improve charge transfer efficiency between their surface and the metal layer. The biosensor achieved a sensitivity of 212.42 deg/RIU, a detection accuracy of 0.35 deg^−1^, and a quality factor of 74.79 RIU^−1^ [70] (Figure 6c). Mudgal et al. developed an SPR biosensor for *Pseudomonas* using a Ag/BaTiO_3_/graphene/affinity layer structure. The inclusion of a BaTiO_3_ layer significantly enhanced its sensitivity and detection capability for *Pseudomonas*. This design showed improved performance over previous configurations, demonstrating promise as a bacterial detection tool [71]. Daher et al. developed an SPR biosensor for rapid waterborne bacteria detection by evaluating four configurations and optimizing the silver thickness, affinity layer, and graphene layers, achieving high sensitivities of 221.63/RIU for *E. coli* and 178.12/RIU for *V. cholerae* [72].

### 4.2. Surface Functionalization and Recognition Element Engineering for Enhanced Specificity and Affinity

Innovative functionalization of sensor surfaces and the development of novel recognition elements are crucial for improving specificity and binding affinity. Kaushal et al. developed sugar-capped gold nanorods (AuNRs) as nanobiosensors for foodborne bacteria by capitalizing on the natural sugar-based adhesion properties of bacterial surfaces. The AuNRs were first coated with polyethylene glycol (PEG) and then covalently functionalized with amine-terminated sugars via an EDC coupling reaction to enable selective bacterial binding. These glycoconjugate-functionalized AuNRs were successfully applied in SPR-based detection, demonstrating a promising strategy for identifying foodborne pathogens [73]. Gür et al. developed a surface-imprinted SPR nanosensor to detect *Escherichia coli* (*E. coli*) within a concentration range of 1 × 10^3^ to 0.5 × 10^1^ CFU/mL, using gold nanoparticles (AuNPs) as the recognition element. To enhance the imprinting process, Cu(II) ions were introduced to promote specific interactions between the bacterial cell wall and amine-functionalized AuNPs, resulting in the formation of selective cavities on the sensor surface. This design significantly improved the affinity for *E. coli* due to the preorganization of metal ions, which facilitated targeted binding between the cell wall and Cu(II)-bound AuNPs, thereby enhancing both recognition capability and sensitivity [65] (Figure 6b). Subramanian et al. investigated the role of physical and chemical surface properties on bacterial adhesion in SPR sensing by developing a functionalized gold-based SPR interface. Through electrophoretic deposition, they coated the sensor with reduced graphene oxide (rGO) films and further modified these surfaces with polyethyleneimine (PEI), poly(sodium 4-styrenesulfonate) (PSS), mannose, and lactose to study strain-specific interactions. This work underscores how tailored chemical functionalization of sensor surfaces can elucidate and manipulate microbial adhesion mechanisms, supporting the design of selective SPR interfaces [66]. Galvan et al. developed a sustained surface plasmon resonance (SPR) biosensor integrated with dielectrophoresis (DEP) to significantly enhance bacterial mass transport for improved detection. A key aspect of their functionalization strategy involved the use of dually functional interdigitated electrodes (IDEs) fabricated with 50 nm gold films and featuring fixed electrode gaps with variable widths. The SPR chip surfaces were modified with mannose as a specific recognition element to target the FimH adhesin on *Escherichia coli*, thereby enhancing cellular adhesion. This innovative label-free DEP–SPR approach, leveraging both functionalization and recognition element engineering, achieved a limit of detection (LOD) of approximately 3.0 × 10^2^ CFU/mL for *E. coli*, which is much higher than conventional SPR biosensor [74].

### 4.3. Advanced Sensor Designs and Hybrid Technology Integration for Improved Efficiency and Utility

The detection efficiency of SPR can also be improved by advanced sensor design and integration with other technologies. Banerjee et al. proposed an optical-fiber SPR-based sensor for the detection of pathogenic bacteria. The sensor utilized gold (Au) and gallium nitride (GaN) as the plasmonic sensing layers, which were integrated into nano-grating structures on the surface of a multimode fiber. The fiber was selected based on its core and cladding diameters, which were optimized for the intended light wavelength, and a section of the cladding was modified to create a flat “D-shaped” area. The sensor demonstrated high sensitivity, achieving a maximum sensitivity of 21,276.6 nm/RIU for detecting *E. coli* in drinking water, and was capable of detecting all four target bacteria [75]. Similarly, Roy et al. developed an SPR-based biosensor employing an optical waveguide with multiple metal–dielectric interface configurations. In terms of system integration, a broadband transverse magnetic (TM) light source was introduced through the input section, and a Gaussian pulse was utilized to excite the sensor structure [76]. Taylor et al. developed a wavelength division multiplexing-based SPR sensor for simultaneous detection of multiple bacteria. The sensor configuration employed a custom-built Kretschmann setup with attenuated total reflection (ATR), in which a wavelength-modulated broadband source was used to illuminate the metal interface at two distinct incident angles. Reflectance signals at corresponding SPR wavelengths were collected by a single spectrophotometer. This integrated optical arrangement directly correlated the resonant wavelength shift with changes in the refractive index near the metal surface. The system achieved limits of detection ranging from 3.4 × 10^3^ to 1.2 × 10^5^ CFU/mL in different matrices, demonstrating effective multiplexed bacterial detection within a compact sensing architecture [77]. Moreover, Nagai et al. developed an integrated detection system for foodborne pathogens by combining asymmetric PCR with a portable SPR sensor. The system specifically targeted the nucA gene of *Staphylococcus aureus* (SA), using asymmetric PCR to preferentially amplify the forward (F) primer strand. The amplified products were quantified using high-performance liquid chromatography (HPLC) and fluorescence electrophoresis, and the results were compared with the response of the SPR sensor. This approach provided a direct performance comparison between conventional methods and the SPR-based platform, demonstrating a novel, portable strategy for rapid detection of foodborne bacteria [78].

### 4.4. Novel SPR Methodologies for Sensitivity and Resolution Enhancement

For detecting trace amounts of bacteria, sophisticated signal amplification strategies are essential. Xia et al. developed an SPR biosensor using an autocatalytic multi-component DNAzyme (MNAzyme) for ultrasensitive DNA detection of three pathogenic bacteria. Functioning as both a recognition element and signal amplifier, the MNAzyme performed cleavage on magnetic beads and triggered a hybridization chain reaction (HCR), resulting in the assembly of nanowires on the SPR sensor. This cascade amplification significantly enhanced the SPR response, enabling highly sensitive detection at concentrations as low as 67 CFU/mL for S. aureus, 57 CFU/mL for K. *pneumoniae*, and 61 CFU/mL for *E. coli* [67] (Figure 6d). To address the fundamental limitation of penetration depth in conventional SPR, Shrivastav et al. highlighted the limitations of conventional SPR, particularly its low penetration depths (less than 300 nm), which restrict the effective analysis of larger biomolecules and surface interactions. To overcome this challenge, they developed a “nearly guided” SPR (NGWSPR) structure to enhance sensor performance. By utilizing wavelengths within the optical telecommunication window, the NGWSPR increased penetration depth, thereby improving the efficacy of sensor’s ability to analyze larger biomolecules. This innovation significantly enhanced the figure of merit and broadened the potential applications of SPR for biomolecular analysis [79]. Pereira et al. developed an advanced SPR-based assay that offers enhanced sensitivity, real-time monitoring, and label-free detection for studying the binding interactions of N-SSc5D, Spα, sCD5, and sCD6 with various bacterial species. Their findings revealed that N-SSc5D displayed a binding affinity comparable to Spα when interacting with *Escherichia coli* and *Listeriamonocytogenes*. Additionally, N-SSc5D demonstrated the ability to distinguish between pathogenic strains of *E. coli* (RS218 and IHE3034) and the non-pathogenic laboratory strain *E. coli* BL21(DE3). These results highlight the promising potential of SPR-based assays as effective tools for the rapid and sensitive screening of interactions between immune-related receptors and pathogen-associated molecular patterns (PAMPs) on microorganisms [80].

In methodological improvements, Boulade et al. introduced a resolution-enhanced SPR imaging method to improve the performance of bacterial detection. In conventional approaches, detection was confined to a limited central region of the image, with sensitivity diminishing toward the periphery. Their novel technique broadened the effective detection zone across the entire field of view, enabling quicker identification of positive signals by detecting individual bacteria. This advancement considerably improved the precision of surface kinetics analysis. The enhanced system was capable of detecting single bacteria within a 1.5 mm^2^ area and facilitated simultaneous analysis of various bacteria–antibody interactions [81]. Further simplifying sensor preparation, Esma streamlined an antibody fragmentation technique to immobilize antibody on the an SPR-based biosensor surface for detecting *Salmonella* [82]. Unlike conventional methods, this approach took less than two hours, which was faster and more efficient without the need for surface functionalization steps. The method enhanced the reliability, sensitivity, and selectivity of bacterial detection, offering a significant improvement over conventional techniques. Enrico et al. developed an SPR-based immunosensor for detecting *Legionella pneumophila* through careful antibody immobilization strategies. The success of their immunosensor was attributed to proper antibody orientation on the sensor surface and the incorporation of microfluidic supports. By monitoring dynamic SPR reflectance signals at varying bacterial concentrations, the sensor demonstrated a detection limit of 10^3^ CFU/mL, providing a sensitive method for detecting this pathogen [42].

Detecting foodborne pathogens under real environmental conditions poses far greater challenges than those encountered in controlled laboratory studies. Actual sample matrices are often highly complex and require standardized sample pretreatment and target bacterial enrichment procedures to effectively eliminate interfering substances and enhance detection sensitivity, thereby ensuring the accuracy and reliability of the final results. Before employing SPR technology for the detection of foodborne pathogenic bacteria of real samples, sample pretreatment or enrichment is typically required. For different matrices such as vegetables, fruits, dairy products, and meat, sterile and clean sampling tools (e.g., scissors, tweezers, sampling spoons) should be used to collect appropriate amounts of samples, which are then placed in dry, clean, and sterile containers. Subsequently, a sterile diluent or enrichment broth is added, and the samples are thoroughly homogenized. The resulting homogenate can be directly subjected to SPR detection, or alternatively, it can first be cultured in a liquid growth medium to increase the bacterial concentration prior to SPR analysis. In addition, food samples can also be immersed in a corresponding sterile diluent, after which the immersion liquid is transferred into a culture medium for further cultivation and enrichment, followed by SPR detection [23,83].

## 5. Advantages and Disadvantages of SPR in Foodborne Pathogen Detection

### 5.1. Advantages of SPR in Foodborne Pathogen Detection

SPR technology enables real-time monitoring of interactions between ligands and receptors. By detecting changes in reflection intensity or resonance angle, it can quantitatively reflect the binding kinetics [84]. This high temporal resolution monitoring makes SPR an ideal tool for studying molecular interactions, particularly in the detection of foodborne pathogens, as it provides rapid and continuous reaction data. A significant advantage of SPR technology is the simplicity of analysis it offers, without requiring pre-labeling or complex sample preparation. Specifically, unpurified samples can be analyzed directly, without the need for conventional chemical labeling steps or sample separation. This label-free technique eliminates the interfering substrate and enables non-destructive detection to effectively preserve the activity of the sample, which is particularly important for biological analytes. Additionally, the high sensitivity and label-free nature of SPR technology result in extremely low background interference. This not only makes SPR particularly suitable for the detection of trace-level targets such as foodborne pathogens, but also significantly ensures the accuracy and reliability of the detection results. At the same time, due to the detection limit of SPR biosensors reaching as low as 10^−9^ mol/L, the surveillance of pathogens or other target molecules at very low concentrations can be easily resolved. This advantage makes SPR a powerful tool for detecting foodborne pathogens, viruses, and other microorganisms, making it especially suitable for early screening and environmental monitoring. SPR biosensors can simultaneously analyze multiple different biological samples in parallel without the need to process each sample individually. This multiplexing capability makes SPR highly suitable for high-throughput detection, significantly improving detection efficiency, especially in complex samples and multi-component analyses [85]. Unlike other conventional detection methods, SPR technology allows real-time monitoring of dynamic changes during reactions, including molecular binding, dissociation, and affinity changes [32]. This provides intuitive and comprehensive data for studying molecular interactions, enables the observation of even fine changes during the reaction process in real-time, and offers richer reaction information than other techniques, making it particularly suitable for dynamic monitoring of complex biological samples.

### 5.2. Disadvantages of SPR in Foodborne Pathogen Detection

Despite its widespread use and significant advantages, SPR technology still exhibits certain limitations in the detection of foodborne pathogens. These challenges arise both from the biological recognition elements commonly employed, such as antibodies, and from inherent physical constraints of the SPR technique itself.

Although antibodies remain crucial recognition molecules in many SPR biosensors due to their specificity, they present several practical limitations. Antibody preparation requires a complex and time-consuming screening process, leading to high production costs. Furthermore, antibodies are typically immobilized through irreversible coupling, which—combined with their sensitivity to environmental conditions such as temperature and pH—often results in gradual inactivation or detachment after repeated washing and regeneration cycles. This not only compromises detection reliability and sensor reusability but also introduces significant batch-to-batch variability, further increasing experimental costs.

Beyond these issues related to recognition elements, SPR technology itself possesses inherent limitations. Temperature, for instance, significantly influences SPR performance, especially in nucleic acid-based detection where hybridization efficiency is highly temperature-dependent. This necessitates precise thermal control to ensure assay accuracy. More fundamentally, the evanescent wave central to SPR sensing has a limited penetration depth of approximately 300 nm. Since bacterial cells are typically several micrometers in size, only a fraction of the captured pathogen lies within the detectable range. This physical constraint inherently reduces the sensitivity and accuracy of direct SPR detection methods for large microbial targets.

When sensor chips of SPR based on surface-modified recognition groups (such as antibodies, aptamers, proteins, and inorganic or organic molecules) are used for the detection of pathogenic bacteria, their surfaces tend to undergo physical or chemical adsorption with the bacteria themselves or with their intracellular components (such as DNA or RNA) [86,87]. This adsorption can occupy active recognition sites and thereby compromise detection accuracy [88]. This issue is particularly pronounced when analyzing protein-rich food matrices or samples with strong acidity or alkalinity. When the recognition molecules are biologically active antibodies or proteins, nonspecific adsorption can easily occur through intermolecular forces such as electrostatic interactions and hydrogen bonding with other proteins [35,56]. Moreover, extreme pH conditions may cause denaturation or deactivation of these recognition molecules, further interfering with detection results. Therefore, minimizing nonspecific adsorption and maintaining the bioactivity of surface-immobilized recognition molecules are critical challenges in the practical application of such sensors. To address this problem, two main strategies are commonly employed. The first involves regeneration treatments to restore the original performance of the sensor surface—for example, rinsing with solutions such as PBST buffer, 5 mM glycine, or 10 mM NaCl until the sensor’s baseline signal returns to stability [89,90,91]. The second strategy is to replace the sensor chip with a new one after each detection cycle. However, both approaches have inherent limitations in terms of regeneration efficiency, detection consistency, and cost control.

Conventional SPR and other optical biosensors generally face challenges in effectively distinguishing between live, dead, and dormant bacteria. Their detection principles rely on changes in the refractive index of the sensing interface medium. However, live and dead bacteria are highly similar in terms of physical dimensions and basic chemical composition [92,93]. Both can bind to the sensor surface and induce refractive index changes, making it difficult to accurately determine bacterial viability based solely on a single response signal. Nevertheless, live bacteria retain intact biological functions, and their surface protein structures and activities are typically well preserved [94,95]. In contrast, the surface proteins of dead bacteria tend to undergo denaturation, degradation, or detachment, thereby losing their ability to bind specifically to ligands [96]. Based on this difference, dead bacteria are less likely to engage in specific binding events on the SPR sensor surface and thus fail to generate a corresponding response signal.To enhance the capability of SPR sensors in discriminating bacterial viability, several strategies can be considered: (1) Pre-labeling with live/dead bacterial stains: Samples can be pretreated with viability-indicating dyes (such as nucleic acid stains or membrane integrity indicators) [97]. The refractive index changes induced by dye-bound bacteria on the SPR sensing interface will differ from those caused by unstained cells, thereby enabling signal-level differentiation between live and dead bacteria. (2) Detection of bacterial metabolites: Only live bacteria are capable of metabolic activity and can release specific metabolites (such as toxins or enzymes) [98]. By using SPR sensors to capture these metabolites, the presence and activity of live bacteria can be indirectly monitored [99]. Variations in metabolite concentrations will correspondingly alter the SPR response signal, enabling specific detection of viable bacterial cells.

## 6. Conclusions and Future Perspectives

This paper systematically elaborates on the detection principles, chip structure, and corresponding detection methods of pathogenic bacteria SPR biosensors. Through a literature review and comparison with other detection methods, it is evident that the SPR technique still has some limitations in detection applications. While SPR biosensors have significant advantages, including ease of operation, no need for sample pretreatment, low detection limits, and label-free characteristics, there is still room for improvement. To further enhance the performance of SPR methods, combining them with other techniques including electrochemical methods and mass spectrometry can significantly expand the application scope of SPR, especially in DNA sequencing analysis and immune specificity recognition. Developing portable, multifunctional chips will enable rapid detection of different pathogens, enabling more convenient and efficient detection in the future. In addition, the advancement of nanotechnology and bioconjugation technologies will substantially improve the performance of SPR in detecting pathogens. Although the current application scope of commercially available SPR sensors is primarily based in research and laboratories, further improvement in cost-effectiveness, user-friendliness, and stability could provide guidance on future development and broaden the application field of SPR sensors.

The introduction of localized surface plasmon resonance (LSPR) technology has provided a new direction for the development of SPR technology [100]. LSPR is a novel SPR sensing technique, wherein collective oscillations of electrons are resonated in cases where the diameter of the metal nanoparticles is smaller than the wavelength of the incident light. The resonance frequency of LSPR is closely related to factors including the charge distribution, shape, and effective electron mass of the metal nanoparticles, and its evanescent wave is generated by the light scattering of the metal nanoparticles. LSPR is a physical optical phenomenon that arises from the interaction between incident light and the free electrons on the surface of metallic nanoparticles. When the frequency of the incident light matches the collective oscillation frequency of the free electrons within the metal nanoparticles, resonance occurs, leading to a significant enhancement of the localized electromagnetic field near the nanoparticle surface. Macroscopically, this manifests as a strong absorption of light within specific wavelength bands by the metallic nanoparticles. The resonance frequency is closely related to factors such as electron density, effective mass, and the shape and size of the nanoparticles [101,102]. In terms of LSPR substrate materials, noble metal colloidal nanoparticles are frequently employed due to their strong plasmonic absorption bands and readily modifiable surfaces, which can be functionalized with biomolecules of high affinity to enhance detection specificity. Metal nanorods, owing to their highly asymmetric structures, exhibit distinct plasmonic resonance modes along their longitudinal and transverse axes, producing composite absorption spectra suitable for simultaneous detection of multiple biological targets [103]. Furthermore, core–shell composite nanoparticles, typically consisting of a dielectric core and a metallic shell, are also widely utilized as LSPR substrates. Their optical properties can be tuned across the visible to near-infrared spectral range by adjusting the core-to-shell size ratio.Studies have further revealed that nanoparticle morphology exerts a pronounced influence on resonance wavelength: sharper geometrical features tend to cause a redshift of the resonance peak. In sensing applications, Orii et al. immobilized gold nanoparticles onto the surface of an indium tin oxide (ITO)-coated multimode optical fiber through electrostatic self-assembly. They observed that analyte-induced electrochemical reactions caused measurable shifts in the LSPR peak position, which exhibited a linear correlation with analyte concentration, enabling simultaneous detection of multiple electroactive species [104]. Yu et al. demonstrated that strong coupling effects between adjacent nanoscale protrusions on roughened silver nanorings could produce pronounced LSPR enhancement, substantially amplifying surface-enhanced Raman scattering (SERS) signals and yielding excellent performance in biosensing applications [105]. Similarly, Nanda et al. reported that LSPR could effectively amplify SERS effects, thereby improving chemical specificity, sensitivity, and multiplexing capabilities [106]. Lamba et al. fabricated low-aspect-ratio silver nanoparticle arrays on nanorippled silicon substrates via a sequential deposition method, effectively minimizing LSPR anisotropy and providing an optimized approach for SERS applications [107]. In addition, the LSPR characteristics of doped metal oxide nanocrystals (such as ZnO, CdO, In_2_O_3_, and WO_3_) have been shown to be tunable within the infrared region, offering new opportunities for the development of next-generation nanocrystal-based optoelectronic devices and sensors [108]. Despite the remarkable progress achieved in laboratory studies of LSPR sensors, their commercialization still faces multiple challenges. These include the need for highly reproducible and morphologically controlled synthesis of metallic nanoparticles; maintaining structural and functional stability under sensing conditions; preventing deactivation or denaturation of biomolecular probes during immobilization on nanoparticle surfaces; and improving sensor reusability and surface regeneration techniques. Further research in these areas is essential to advance the practical application and scalability of LSPR-based sensing technologies. Compared to conventional SPR technology, LSPR does not require complex optical systems, making it highly promising, particularly for applications in rapid and simple detection fields. We also systematically summarized the relevant characteristics of LSPR and SPR (as shown in Table 3). LSPR exhibits significant differences from conventional SPR in terms of its occurrence location, plasmonic modes, and excitation mechanisms. LSPR offers a much stronger localized electromagnetic field enhancement effect, and its resonance wavelength can be flexibly tuned by adjusting the size and morphology of nanostructures. This tunability makes it highly suitable for a wide range of functional structural designs. In addition, LSPR technology possesses remarkable potential for miniaturization and can be easily integrated into portable detection platforms, thereby improving the practicality and efficiency of on-site testing. Moreover, by combining LSPR sensors with emerging technologies such as microfluidics and smartphone-based optical readout systems, rapid, low-cost, and highly sensitive detection of pathogenic bacteria can be achieved [83,109]. Consequently, LSPR has been recognized as a key and promising development direction in the field of foodborne pathogen sensing.

Microfluidic technology is another important technology driving the improvement of biosensors [23,110]. By miniaturizing fluid flow and mass transfer processes to the micron or nanoscale, microfluidic technology enables sample preparation, capture, detection, and other processes to be performed on a single integrated platform. Biosensors based on microfluidic technology can convert biomolecular recognition events into measurable physical or chemical signals, offering advantages, including low consumption of samples and reagents, flexibility in operation, high integration, and short detection times. Additionally, microfluidic platforms can be integrated with optical, electrical, magnetic, and other technologies, greatly enhancing detection efficiency and driving the development of rapid and convenient pathogen detection applications [111,112].

Conventional bacterial isolation, culturing, and biochemical identification methods can no longer meet the urgent demands for rapid pathogen detection. The application of SPR technology in rapid pathogen diagnosis offers a new possibility. Compared to traditional PCR methods, which require designing different target sequences and primers for corresponding pathogens and establishing multiple amplification systems, SPR reduces the workload and increases detection throughput. Although SPR technology has shown excellent performance in pathogen detection, it currently does not cover all pathogen information, and its detection range is relatively narrow. As a result, it may not be able to provide accurate results for certain clinical samples. Therefore, future SPR biosensor detection systems should integrate genomic DNA information of clinically relevant pathogenic microorganisms to expand the detection range and improve accuracy and sensitivity.

## Figures and Tables

**Table 1:** Comparison of major microbial detection technologies.

Technology Category	Detection Principle	Time Required	Advantages	Disadvantages	Ref.
Traditional Culture-Based Methods	Microbe isolation, culture, and identification	3–7 days	Gold standard, accurate, quantitative, low cost	Time-consuming, labor-intensive, cannot detect VBNC states	[17]
Immunological Methods	Antigen–antibody reaction	Minutes–Hours	Rapid, simple, suitable for on-site screening	Potential false positives/negatives, difficult to quantify, cannot distinguish live/dead bacteria	[18]
Molecular Biology Methods	Amplification of specific gene fragments	Several Hours	Highly sensitive and specific, rapid, can detect hard-to-culture bacteria	Cannot distinguish live/dead bacteria, prone to contamination, expensive equipment	[19]
Metabolic/Phenotypic Methods	Detection of metabolic products or physical changes	Hours–2 Days	Detects live bacteria, can be automated	Difficult for direct identification, limited sensitivity, requires databases	[20]
Biosensors	Biological recognition + signal transduction	Minutes–30 min	Extremely fast, highly sensitive, portable, potential for real-time monitoring	Immature technology, poor stability, susceptible to interference	[21]
Whole Genome Sequencing	Determination of the entire genome sequence	1–3 Days	Most comprehensive information, highest resolution, precise traceability	High cost, complex data analysis, time-consuming	[22]
SPR	Refractive index change from biomolecular binding	Minutes–1 h	Label-free, real-time, and dynamic monitoring, fast detection, minimal sample volume, potential for automation and high-throughput	Expensive equipment, cannot distinguish live/dead bacteria	[23]

**Table 2:** Performance comparison of various SPR sensors in foodborne pathogen detection.

	LOD (Limit of Detection)	Linear Range	Foodborne Pathogens	Ref.
Direct detection	10^3^ CFU/mL	0–10^5^ CFU/mL	*E. coli*	[36]
	50 CFU/mL	50–10^8^ CFU/mL	*Staphylococcus aureus*	[37]
	57 CFU/mL	1.5 × 10^1^–1.5 × 10^7^ CFU/mL	*Salmonella* sp.	[38]
	94 CFU/mL	1000–8000 CFU/mL	*E. coli*	[39]
	14 CFU/mL	1.4 × 10^1^–1.4 × 10^9^ CFU/mL	*Salmonella enteritidis*	[40]
	43 CFU/mL	10^2^–10^5^ CFU/mL	*Legionella pneumophila*	[42]
	0.9984 CFU/mL	3.7 × 10^5^–3.7 × 10^9^ CFU/mL	*Vibrio cholerae O1*	[45]
	10^4^ CFU/mL	5 × 10^4^–5 × 10^7^ CFU/mL	*S. typhimurium*	[46]
	10 CFU/ml	10^1^–10^3^ CFU/mL.	*Legionella pneumophila*	[49]
	10^5^ CFU/mL	10^5^–10^8^ CFU/mL	*Salmonella*	[54]
	10^4^ CFU/mL	10^2^–10^6^ CFU/mL	*Salmonella*	[55]
	2 CFU/mL	100–10^8^ CFU/mL	*Salmonella*	[61]
Indirect detection	131 CFU/mL	5–5 × 10^6^ CFU/mL	*Campylobacter jejuni*	[64]
	1 CFU/mL	0.5 × 10^1^–1 × 10^3^ CFU/mL	*E. coli*	[65]
	10^2^ CFU/mL	10^2^–10^9^ CFU/mL	*E. coli*	[66]
	57 CFU/mL	1 × 10^2^–1 × 10^6^ CFU/mL	nBSI bacteria	[67]

**Table 3:** A comparative analysis of the key characteristics between LSPR and SPR.

Feature	LSPR	SPR
Location	Surface of metal nanoparticles	Surface of continuous, flat metal thin films
Plasmon Mode	Localized oscillation of electron cloud within the 3D nanostructure	Propagating wave along the 2D metal film surface, extending and oscillating at the interface
Excitation Method	Simple, direct excitation (e.g., using broad-spectrum light like white light)	Complex, requires wavevector matching, excited by polarized light at a specific incident angle
Electromagnetic Field Decay	Field intensity decays rapidly from the surface (~10–30 nm)	Longer decay length of the field intensity
Equipment Size and Cost	Relatively simple, miniaturized, lower cost	Complex, requires precise optical components, bulky and expensive equipment
Advantages and Disadvantages	Adv: Simple equipment, easy integration, higher sensitivity to changes immediately at the surface	Adv: Ultra-high sensitivity, can provide precise kinetic data
	Dis: Generally lower absolute sensitivity than SPR, slightly weaker for quantitative kinetic analysis	Dis: Expensive equipment, bulky, more sensitive to bulk effects
